# Low expression of NR1D1 and NR2E3 is associated with advanced features of retinoblastoma

**DOI:** 10.1007/s10792-024-03055-3

**Published:** 2024-03-14

**Authors:** Jie Ding, Jie Sun, Rui-Qi Ma, Ke Zheng, Yi-Nan Han

**Affiliations:** Department of Ophthalmology, Eye & ENT Hospital of Fudan University, 83 Fenyang Road, Xuhui District, Shanghai, 200031 China

**Keywords:** Retinoblastoma, NR1D1, NR2E3, Orphan nuclear receptors, High-risk pathology

## Abstract

**Purpose:**

To investigate the expression of nuclear receptor subfamily 1 group D member 1 (NR1D1) and nuclear receptor subfamily 2 group E Member 3 (NR2E3) in retinoblastoma (RB) and their correlation with the clinical and pathological features of RB.

**Methods:**

Immunohistochemical (IHC) assays were performed to detect and evaluate the expression levels of NR1D1 and NR2E3 in paraffin-embedded tissue samples. The relationship between the expression levels and clinicopathological characteristics of RB patients was analyzed using the χ^2^ test or Fisher exact test.

**Results:**

A total of 51 RB patients were involved in this research. The expression levels of NR1D1 (*P* = 0.004) and NR2E3 (*P* = 0.024) were significantly lower in RB tumor tissues than in normal retina. The expression levels of NR1D1 and NR2E3 were less positive in RB patients with advanced stages (*P* = 0.007, *P* = 0.015), choroidal infiltration (*P* = 0.003, *P* = 0.029), and optic nerve infiltration (*P* = 0.036, *P* = 0.003). In addition, a low expression level of NR2E3 was associated with high-risk pathology (*P* = 0.025) and necrosis (*P* = 0.035) of RB tissues.

**Conclusion:**

The expression levels of NR1D1 and NR2E3 were decreased in RB and closely associated with the clinical stage and high invasion of the disease. These findings provide new insights into the mechanism of RB progression and suggest that NR1D1 and NR2E3 could be potential targets for treatment strategies.

## Introduction

Retinoblastoma (RB) is the most common pediatric intraocular malignancy, which affects one in every 16 000 to 18 000 children [1]. Nearly 95% of RB patients are diagnosed before 5 years old [2]. The majority of RB patients have a biallelic deletion of the RB1 gene, which is proven to be essential for the onset and development of RB [3]. Despite the significant improvement in the survival rate of RB due to current treatments including intra-arterial chemotherapy and enucleation surgery, the prognosis remains challenging for cases with extensive choroidal and optic nerve invasion. Additional treatment strategies are needed to improve outcomes in cases with significant invasion. Therefore, investigating the invasion features of RB is crucial for identifying potential therapeutic options.

Nuclear receptor subfamily 1 group D member 1 (NR1D1) and nuclear receptor subfamily 2 group E member 3 (NR2E3) are orphan nuclear receptors (ONRs) that are ubiquitously expressed and are involved in essential biological processes, such as embryogenesis, differentiation, and homeostasis maintenance [4]. As a core component of the cellular circadian clock system, ONRs coordinate a variety of intricate networks in vivo and impact the progression of diseases, especially cancers. Recently, the role of ONRs in human malignancies has received increasing attention. It has been demonstrated that NR1D1, also known as REV-ERB-α, inhibits the growth of ovarian cancer and is related to the prognosis of breast cancer [5, 6]. NR2E3 has previously been defined as a photoreceptor-specific nuclear receptor, acting as an essential character in the development of normal retina as well as in the suppression of liver and breast cancer [7, 8]. These findings suggest that NR1D1 and NR2E3 may play critical roles in tumorigenesis and cancer progression, highlighting their therapeutic relevance. Yet, it remains unclear how these two ONRs, NR1D1 and NR2E3, affect the progression of RB.

In this research, we conveyed immunohistochemical (IHC) staining to examine the expression of NR1D1 and NR2E3 in the tumor tissues of RB patients, and explored their correlation with clinical and pathological features of RB, including disease stages, infiltration of the choroid and optic nerve.

## Materials and methods

### Sample size calculation

Sample size calculation was conducted using PASS software. The positivity rate of IHC in RB tumors was set at 50% based on our preliminary experimental results. The significance level (α) was chosen as 0.05, and the power (1-β) was set to 0.9. The enrollment ratio was set at 1:4. According to these parameters, the estimated sample size was approximately 51, with 10 cases in the control group.

### Patient and samples

Paraffin-embedded sections of 51 RB tissues and 12 normal retina tissues were obtained from the Eye and ENT Hospital of Fudan University between 2018 and 2023. The normal retina samples were obtained from patients who have undergone orbital exenteration due to malignancies of the eyelid or orbit without involvement of the eyeball. All the RB patients received enucleation of the affected eyes without any prior treatment. The diagnosis of RB was made based on the pathologic observation, and the classification of RB was made according to the International Intraocular Retinoblastoma Classify (IIRC) [9]. High-risk pathologic features were defined as pT3 and pT4 categories in the 8th edition of the American Joint Committee on Cancer (AJCC) [10]. pT3 was defined as RB with massive choroidal invasion (> 3 mm in largest diameter, multiple foci of focal choroidal involvement totaling > 3 mm, or any full-thickness choroidal involvement), retrolaminar invasion of the optic nerve head not involving the transected end of the optic nerve, any partial-thickness involvement of the sclera within the inner two thirds or full-thickness invasion into the outer third of the sclera, invasion into or around emissary channels. pT4 was defined as RB with evidence of extraocular tumor (tumor at the transected end of the optic nerve, tumor in the meningeal spaces around the optic nerve, full-thickness invasion of the sclera with invasion of the episclera, adjacent adipose tissue, extraocular muscle, bone, conjunctiva, or eyelids).

Demographic and clinical data including age, gender, laterality, and enucleated eye were collected, and histopathologic features such as infiltration of choroid, infiltration of optic nerve, cell differentiation, tumor calcification, and necrosis were recorded. All participants provided written informed consent, and the approval of the institutional ethics committee was obtained.

### Immunohistochemical staining

Paraffin-embedded tissue sections of 4 μm thickness were firstly heated at 60 °C for 30 min, then deparaffinized and rehydrated using a series of xylene and graded ethanol. After antigen repair in sodium citrate solution through microwave heating, the slides were then incubated with 3% hydrogen peroxide for 15 min to block the activity of endogenous peroxidases. Next, 1 h incubation with 5% bovine serum albumin/TBST (20 mmol/L Tris, pH 7.5,150 mmol/L NaCl, 0.1% Tween 20) at room temperature was performed to block nonspecific protein. After that, the slides were incubated with primary antibodies overnight at 4 °C, followed by incubation with horseradish peroxidase (HRP) conjugated secondary antibodies for 1 h at room temperature. DAB Substrate Kit (K-4100, Vector Laboratories) was used to detect the presence of HRP according to the manual’s instructions. Finally, the slides were counterstained with hematoxylin and mounted in Neutral Balsam Mounting Medium.

Primary antibodies used in IHC staining were NR1D1 monoclonal antibody (1:200, H00009572-M02, Abnova) and NR2E3 polyclonal antibody (1:200, 14246-1-AP, Proteintech). According secondary antibodies were HRP-labeled Goat Anti-Mouse IgG(H + L) (1:100, A0216, Beyotime) and HRP-labeled Goat Anti-Rabbit IgG(H + L) (1:100, A0208, Beyotime).

### Immunohistochemical staining analysis

The stained slides were analyzed using ImageJ software. Integrated Optical Density (IOD) and Area of staining region in each slide were calculated, and the relative quantified expression level of staining slides was shown as Average Optical Density (AOD, AOD = IOD/Area). The high and low positive groups were defined according to a cutoff value set as the median of AOD, with the AOD of the high positive group being equal to or higher than the median and the low positive group being lower.

### Statistical analysis

Statistical analyses were performed and graphics were generated using SPSS 22.0 and GraphPad Prism 8 software. Statistical differences between patient groups and controls were analyzed by unpaired two-tailed Student’s t-test. The χ^2^ test or Fisher exact test was used for categorical variables as appropriate. A *P* value < 0.05 was considered significant.

## Results

### Clinical and pathological features of RB patients

A total of 51 enucleated eyes from 51 RB patients were included in this study, including 24 males (47.06%) and 27 females (52.94%) with a mean age of 26.37 ± 14.96 months. Unilateral lesions were observed in 49 patients (96.08%), with 2 (3.92%) patients having bilateral lesions. Among these, 22 (43.14%) patients underwent right eye enucleation, and 29 (56.86%) patients underwent left eye surgery. According to the IIRC classification, 21 (41.18%) patients were classified as group D, while 30 (58.82%) were classified as group E. High-risk pathological features were observed in 33 (64.71%) patients. Infiltration of choroid was observed in 20 (39.22%) patients, and infiltration of optic nerve was present in 28 (54.90%) patients. RB cell differentiation type was good or moderate in 11(21.57%) patients, while 40 (78.43%) patients had poor differentiation. Calcification was found in the RB tissues of 19 (37.25%) patients, while necrosis was found in 30 (58.82%) patients. The clinical and pathological features of RB patients are summarized in Table 1.

**Table 1:** Clinical and pathological features of RB patients

Variable	RB cases (%)	Variable	RB cases (%)
Age (month, mean ± SD)	26.37 ± 14.96	Infiltration of choroid	
Gender		Yes	20 (39.22%)
Male	24 (47.06%)	No	31 (60.78%)
Female	27 (52.94%)	Infiltration of optic nerve	
Laterality		Yes	28 (54.90%)
Unilateral	49 (96.08%)	No	23 (45.10%)
Bilateral	2 (3.92%)	Differentiation	
Enucleated eye		Good or moderate	11 (21.57%)
Right	22 (43.14%)	Poor	40 (78.43%)
Left	29 (56.86%)	Calcification	
Stage		Yes	19 (37.25%)
Group D	21 (41.18%)	No	32 (62.75%)
Group E	30 (58.82%)	Necrosis	
High-risk pathology		Yes	30 (58.82%)
Yes	33 (64.71%)	No	21 (41.18%)
No	18 (35.29%)		

*RB* retinoblastoma

### Lower expression level of NR1D1 and NR2E3 in RB compared to normal retina

**Fig. 1:**
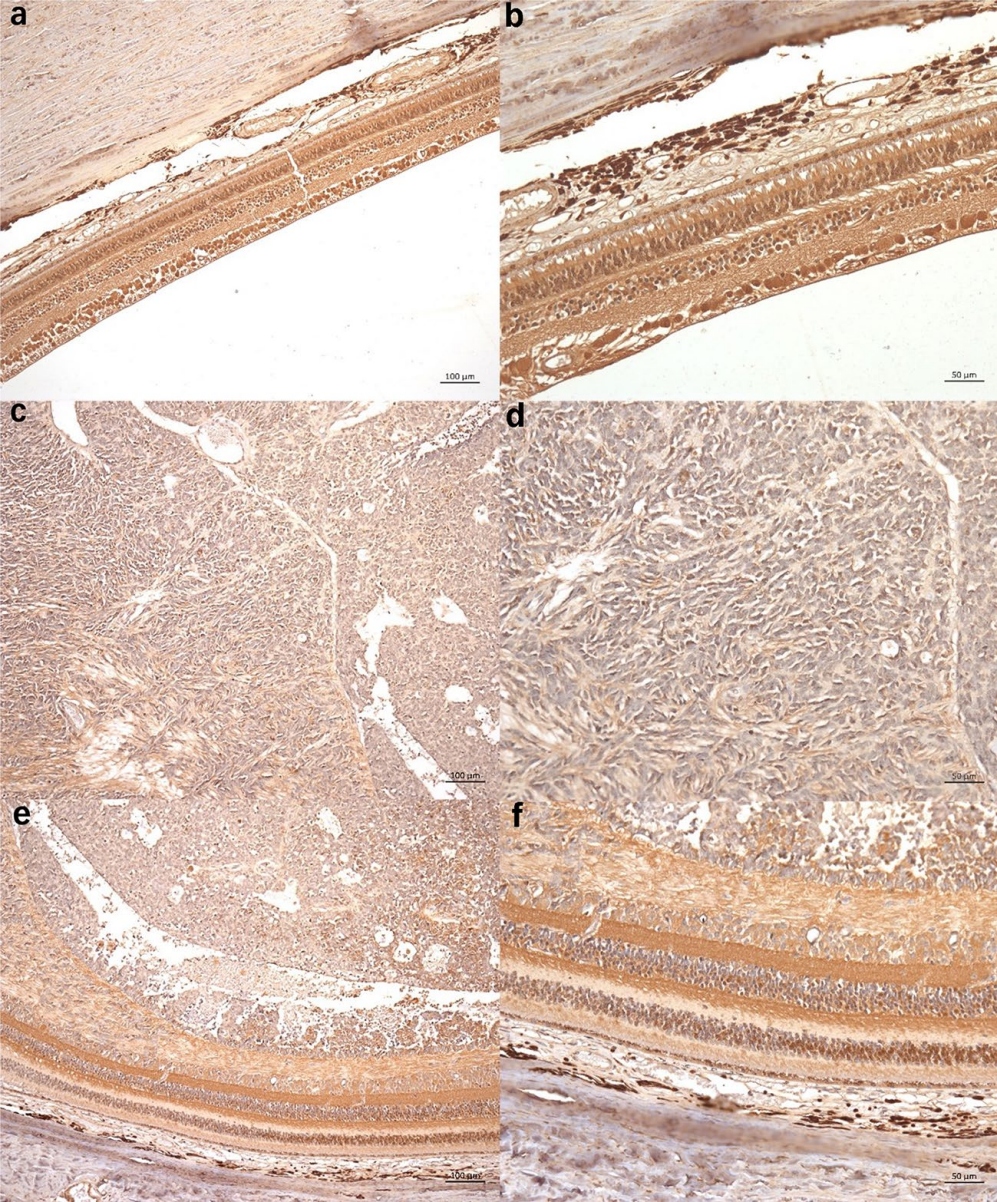
Representative photomicrographs of IHC expression of NR1D1: normal retina (**a**, **b**), RB tumor (**c**, **d**), and non-tumor retina in RB patients (**e**, **f**). Scale bar, 100 μm (100 ×), 50 μm (200 ×)

**Fig. 2:**
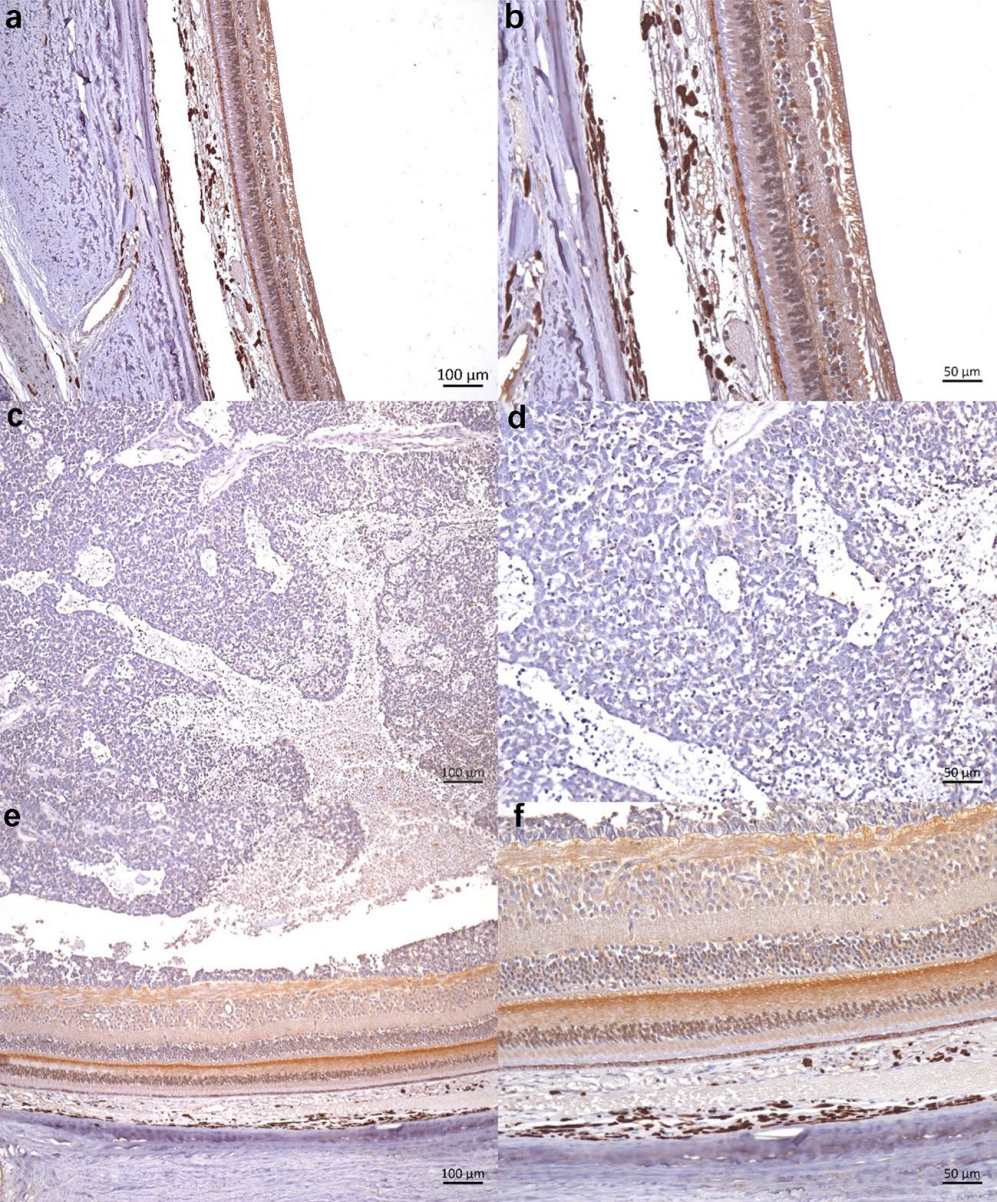
Representative photomicrographs of IHC expression of NR2E3: normal retina (**a**, **b**), RB tumor (**c**, **d**), and non-tumor retina in RB patients (**e**, **f**). Scale bar, 100 μm (100 ×), 50 μm (200 ×)

**Fig. 3:**
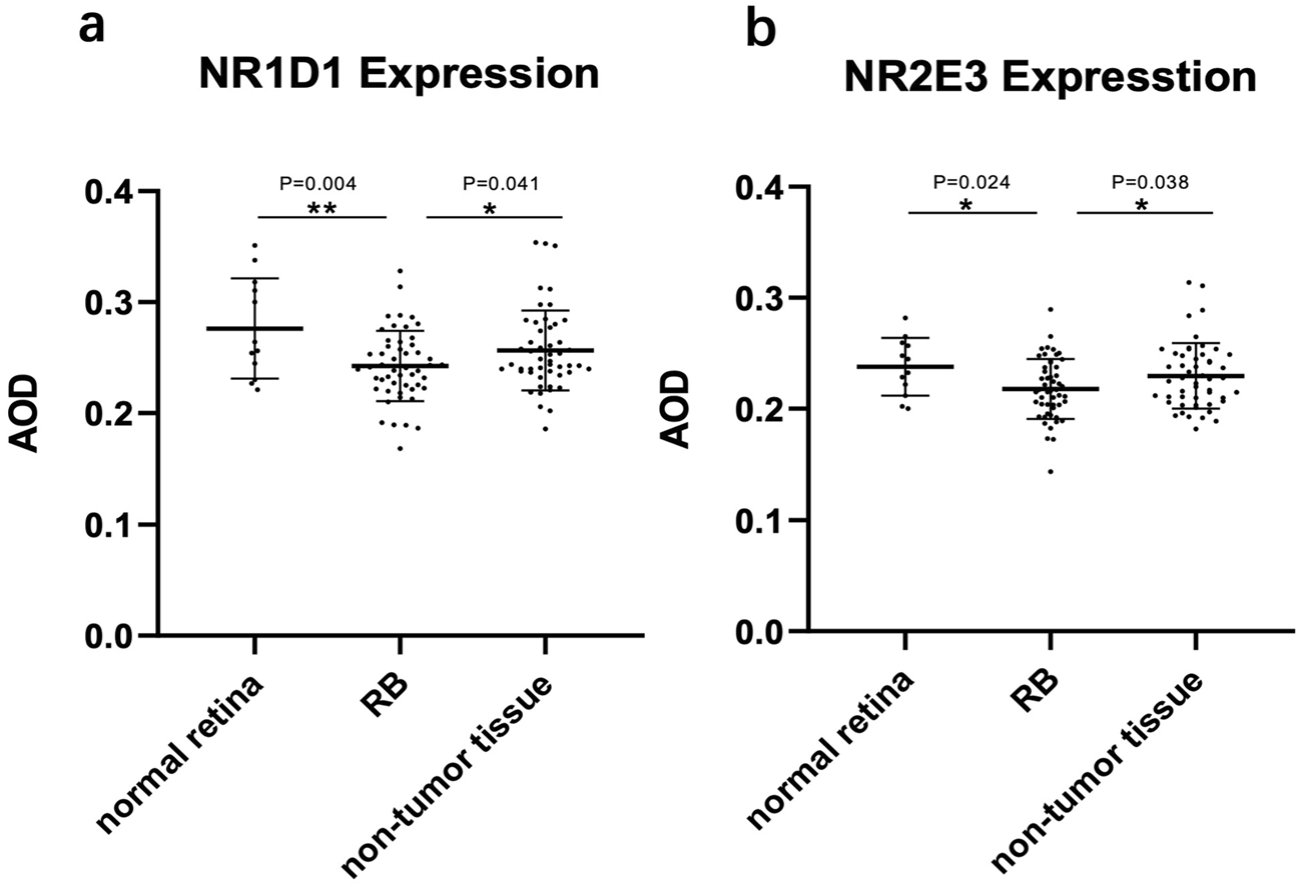
AOD of IHC staining: AOD of NR1D1 (**a**) and NR2E3 (**b**) in tissue sections of normal retina, RB tumor and non-tumor retina

In order to investigate the expression level of NR1D1 and NR2E3 in RB patients, IHC staining and image analysis were performed in 51 RB tissues and 12 normal controls. Representative pictures are shown in Figs. 1 and 2. In normal retina and non-tumor retina tissues, NR1D1 (Fig. 1) and NR2E3 (Fig. 2) were expressed in the cell nucleus of the outer nuclear layer, inner nuclear layer, and ganglion cell layer, especially high-expressed in the outer nuclear layer. However, NR1D1 and NR2E3 were less positive in the cell nucleus of RB tissues. For NR1D1, the average AOD value of RB tissues is 0.243 ± 0.031, which is significantly lower than the average AOD of 0.276 ± 0.043 in normal retina (*P* = 0.004), while the AOD of NR1D1 in the adjacent non-tumor retina of RB patients (0.256 ± 0.036) was not significantly different from the AOD of normal retina (*P* = 0.108) but higher than that of RB tissue (*P* = 0.041) (Fig. 3a). Similar results were achieved in NR2E3 staining, which showed the average AOD of 0.218 ± 0.027, 0.238 ± 0.025, and 0.230 ± 0.029 in RB tissue, normal retina, and non-tumor retina in RB patients, respectively. The expression level of NR2E3 was significantly lower in RB tissues than in normal retina (*P* = 0.024 and non-tumor retina (*P* = 0.038), and no difference of AOD was shown between normal retina controls and non-tumor retina tissues (*P* = 0.386). (Fig. 3b).

### Correlation of NR1D1 and NR2E3 expression levels with clinicopathological features of RB patients

The χ^2^ test and Fisher exact test were used to investigate the relationship between the expression levels of NR1D1 and NR2E3 and clinicopathological characteristics of RB patients, with high and low positive groups defined based on the median of AOD. Out of the 51 RB patients included in this study, 25 patients had high expression of NR1D1 while 26 had low expression. For NR2E3, the high-positive group involved 26 RB patients, and the low-expression group involved 25 RB patients. There was no significant difference in age, gender, laterality, enucleated eye, differentiation, or calcification between the high and low expression groups of NR1D1 and NR2E3 (Tables 2 and 3). Statistical significance was found in the IIRC stage (*P* = 0.007), infiltration of choroid(*P* = 0.029), and infiltration of optic nerve (*P* = 0.036) between high and low expression of NR1D1 (Table 2). In groups of NR2E3, expression level showed significant differences in the IIRC stage (*P* = 0.015), high-risk pathology (*P* = 0.025), infiltration of choroid (*P* = 0.003), infiltration of optic nerve(*P* = 0.003) and necrosis (*P* = 0.035) (Table 3). Low levels of both NR1D1 and NR2E3 were observed in the majority of RB patients with group E (66.67%, 63.33%), infiltration of choroid (70.00%, 75.00%), and infiltration of optic nerve (64.29%, 67.86%). In addition, 20 (60.61%) RB patients with high-risk pathology were detected with a low expression level of NR2E3. To conclude, low expression of both NR1D1 and NR2E3 was associated with advanced features of RB.

**Table 2:** Correlation of NR1D1 expression level with clinicopathological features of RB patients

Variable	High expression	Low expression	*P* value
Age			
> 2 years old	7	8	
≤ 2 years old	18	18	
Total	25	26	0.828
Gender			
Male	11	13	
Female	14	13	
Total	25	26	0.668
Laterality			
Unilateral	24	25	
Bilateral	1	1	
Total	25	26	0.977
Enucleated eye			
Right	10	12	
Left	15	14	
Total	25	26	0.657
IIRC stage			
Group D	15	6	
Group E	10	20	
Total	25	26	0.007*
High-risk pathology			
Yes	13	20	
No	12	6	
Total	25	26	0.063
Infiltration of choroid			
Yes	6	14	
No	19	12	
Total	25	26	0.029*
Infiltration of optic nerve			
Yes	10	18	
No	15	8	
Total	25	26	0.036*
Differentiation			
Good or moderate	20	20	
Poor	5	6	
Total	25	26	0.789
Calcification			
Yes	10	9	
No	15	17	
Total	25	26	0.691
Necrosis			
Yes	14	16	
No	11	10	
Total	25	26	0.688

*NR1D1* nuclear receptor subfamily 1 group D member 1, *IIRC* international intraocular retinoblastoma classify *RB* retinoblastoma

Statistical analyses were performed by the χ2 test

*P < 0.05 was considered statistically significant

Statistically significant numbers in bold

**Table 3:** Correlation of NR2E3 expression level with clinicopathological features of RB patient

Variable	High expression	Low expression	*P* value
Age			
> 2 years old	8	7	
≤ 2 years old	18	18	
Total	26	25	0.828
Gender			
Male	14	10	
Female	12	15	
Total	26	25	0.322
Laterality			
Unilateral	25	24	
Bilateral	1	1	
Total	26	25	0.977
Enucleated eye			
Right	9	13	
Left	17	12	
Total	26	25	0.210
IIRC stage			
Group D	15	6	
Group E	11	19	
Total	26	25	0.015*
High-risk pathology			
Yes	13	20	
No	13	5	
Total	26	25	0.025*
Infiltration of choroid			
Yes	5	15	
No	21	10	
Total	26	25	0.003*
Infiltration of optic nerve			
Yes	9	19	
No	17	6	
Total	26	25	0.003*
Differentiation			
Good or moderate	6	5	
Poor	20	20	
Total	26	25	0.789
Calcification			
Yes	12	7	
No	14	18	
Total	26	25	0.180
Necrosis			
Yes	19	11	
No	7	14	
Total	26	25	0.035*

*NR2E3* nuclear receptor subfamily 2 group E member 3, *IIRC* international intraocular retinoblastoma classify, *RB* retinoblastoma

Statistical analyses were performed by the χ2 test

*P < 0.05 was considered statistically significant

Statistically significant numbers in bold

## Discussion

RB is a malignant neoplasm originating from maturing cone precursors, accounting for about 3% of all types of cancer in childhood [11, 12]. Extraocular tissue invasion is the primary feature of RB that can threaten life and vision. The primary goal of RB management is to preserve children’s lives, their globes and vision. Although timely treatment has reduced the mortality of RB, it is still debatable whether globe-preserving treatment increases the risk of extraocular metastasis in advanced RB. Hence, the detection of extraocular invasive features is critical in managing RB. To the best of our knowledge, this is the first study that explored the expression of two ONRs, NR1D1 and NR2E3, in the tumor tissues of RB patients. The expression of NR1D1 and NR2E3 were found to be lower in RB tumor tissues than in normal and non-tumor retinal tissues.

Previously, NR1D1 and NR2E3 were reported to co-express in the outer neuroblastic layer of developing mouse retina and in the outer nuclear layer within rods and cones of adult mouse retina, regulating critical transcriptional networks in the functions of photoreceptors [13]. Our study showed that NR1D1 and NR2E3 were expressed in the cell nucleus of outer nuclear layer, inner nuclear layer, and ganglion cell layer in retinal tissues, with particularly high levels in the outer nuclear layer consisting of photoreceptors. However, the expression level of NR1D1 and NR2E3 was less positive in RB tumor tissues.

The low levels of NR1D1 and NR2E3 expression in RB are consistent with previous findings in other cancers. It was reported that the low expression level of NR1D1 was related to poor prognosis in gastric cancer, and NR1D1 was involved in tumor development through suppressing the apoptosis of cancer cells [14]. In breast cancer, the antiproliferative effects of NR1D1 were achieved by targeting cyclin A2 and interfering with the cancer cell cycle [15]. By suppressing oncogenic drivers including BRAF and HRAS, agonists of NR1D1 were considered to have anticancer properties and offer a prospective therapeutic target [16]. Our study provides evidence for a potential tumor-suppressing function of NR1D1 in RB, based on the novel findings that low expression levels of NR1D1 associated with advanced disease stage and choroidal and optic nerve infiltration.

NR2E3 is predominantly known for its role in the differentiation and development of retinal photoreceptors, which are related to retinal dystrophies [17]. There has been relatively little research on the anti-tumor ability of NR2E3. NR2E3 was considered to regulate the expression of the estrogen receptor in breast cancer cells via binding to its promotor [7]. Khanal et al. reported that NR2E3 acted as a positive upstream transcriptional regulator of aryl hydrocarbon receptor, with their levels being associated with survival outcomes in liver cancer [8]. In the present study, our findings highlight a significant correlation between NR2E3 and advanced stage and high-risk pathological features of RB, including choroidal and optic nerve invasion.

Recent studies have reported that RB derived from less differentiated cones with neuronal or ganglion cell markers showed higher invasion and metastasis compared to mature cone markers [12]. Consistent results were observed in other studies, which pointed out that adverse histopathological features, such as post-laminar optic nerve invasion and deep choroid invasion, were more frequent in RB tumors derived from an early uncommitted cell type [18]. Moreover, hereditary characteristics were found to be different between these two subtypes of RB, with RB of mature cone markers harboring mainly *RB1* gene inactivation and RB of neuronal or ganglion cell markers harboring other genetic alternations like *MYCN* amplification. In our study, NR1D1 and NR2E3, which play a critical role in the development and differentiation of photoreceptors, showed low expression levels in RB tumor tissues compared to retina and were strongly associated with invasive clinicopathological features of RB. However, no significant difference was found between overall levels of cell differentiation in RB and expression of NR1D1 and NR2E3 in our research. Further investigations on a larger sample size are necessary to determine the relationship between NR1D1 and NR2E3 expression and RB heterogeneity or clinical features. Additionally, further exploration of the biological mechanisms underlying RB progression is necessary, particularly to determine if NR1D1 and NR2E3 serve as early indicators of more aggressive RB or if these genes are downregulated as the tumor grows. This may shed new light on the pathogenesis and progression of RB.

## Conclusion

In summary, our study demonstrated a significant reduction in the expression of NR1D1 and NR2E3 in RB tumors compared to normal retina and a strong association between their expression levels, advanced stage and extraocular invasion of RB. Investigation into the molecular mechanisms of NR1D1 and NR2E3 may provide insights into the unique features of choroidal and optic nerve invasion in RB and lead to the development of novel therapeutic targets for RB treatment.
